# Where there is no evidence: implementing family interventions from recommendations in the NICE guideline 11 on challenging behaviour in a South African health service for adults with intellectual disability

**DOI:** 10.1186/s12913-019-3999-z

**Published:** 2019-03-13

**Authors:** Ockert Coetzee, Leslie Swartz, Charlotte Capri, Colleen Adnams

**Affiliations:** 1 Department of Psychiatry and Mental Health, University of Cape Town, J-Block, Groote Schuur Hospital, Observatory, Cape Town, 7925 South Africa; 2 Alan J Flisher Centre for Public Mental Health, Department of Psychology, Stellenbosch University, Private Bag X1, Matieland, 7602 South Africa

**Keywords:** Intellectual disability, Challenging behaviour, NICE guidelines, Low-income contexts

## Abstract

**Background:**

Low- and middle-income countries often lack the fiscal, infrastructural and human resources to conduct evidence-based research; similar constraints may also hinder the application of good clinical practice guidelines based on research findings from high-income countries. While the context of health organizations is increasingly recognized as an important consideration when such guidelines are implemented, there is a paucity of studies that have considered local contexts of resource-scarcity against recommended clinical guidelines.

**Methods:**

This paper sets out to explore the implementation of the NICE Guideline 11 on family interventions when working with persons with intellectual disability and challenging behavior by a group of psychologists employed in a government health facility in Cape Town, South Africa.

**Results:**

In the absence of evidence-based South African research, we argue that aspects of the guidelines, in particular those that informed our ethos and conceptual thinking, could be applied by clinical psychologists in a meaningful manner notwithstanding the relative scarcity of resources.

**Conclusion:**

We have argued that where guidelines such as the NICE Guidelines do not apply contextually throughout, it remains important to retain the principles behind these guidelines in local contexts. Limitations of this study exist in that the data were drawn only from the clinical experience of authors. Some of the implications for future research in resource-constrained contexts such as ours are discussed. Smaller descriptive, qualitative studies are necessary to explore the contextual limitations and resource strengths that exist in low- and middle-income settings, and these studies should be more systematic than drawing only on the clinical experience of authors, as has been done in this study.

## Background

The importance of providing good standards of care for persons with intellectual disability (ID) is increasingly recognized [1, 2]. The publication of a growing number of evidence-based studies and recommendations of good practice, such as the guidelines of the National Institute for Clinical Excellence (NICE), when working with families of people with ID and behavior that challenges, marks the maturity of family and caregiving research in the field of ID. The NICE system is used in the United Kingdom, and guidelines are drawn up using the best available evidence for what has been shown to work in a range of health-related fields [3]. The National Institute for Clinical Excellence has also published guidelines on the prevention, assessment and management of mental health problems among those with ID [4]. Both these guidelines highlight the need to provide family interventions if a person presents with behavior that challenges [3, 4]; in the field of ID in general, best practice suggests involving families and community resources in all aspects of care [5]. The NICE system is not the only set of guidelines available. For example, the Health Equalities Framework is based on an understanding of the inequality of health care access for populations with ID, and measures outcomes across different health indicators which include behaviors that place a person or their carers at risk [6]. A comprehensive analysis of available research on intellectual disability and behavior that challenges has recently been published, specifically insofar as the research findings inform good social work practice [7]. In this article, we focus on the NICE Guideline 11 on behavior that challenges as an example of good clinical practice, firstly because they are comprehensive and well-regarded, and secondly, because these guidelines are widely read and referred to in health service practice in South Africa, where we work.

Ideally, all guidelines should be applied contextually, and it is well established that there may be barriers to the full application of guidelines even in well-resourced contexts [2, 8]. The NICE guideline on the management of behavior that challenges is explicit in its requirements of a contextual understanding of the organizational design and family environments of service-users before individualized interventions are implemented [3]. In clinical practice, it does not make sense to apply guidelines which depend on evidence that does not apply in a particular context. For example, it is incongruent to recommend MRI as a diagnostic tool in countries where there are no MRI machines. The NICE Guidelines are designed for use in the UK, a country in which there are certain standards of care within the health system, as well as expectations regarding how the country context operates. The UK is a stable, industrialized country with a health system which, despite shortcomings, functions efficiently. Were social and economic conditions in the UK greatly different, the NICE Guidelines would reflect that contextual difference in the scope and range of response.

Most families of persons with ID live in low- and middle-income countries, commonly in conditions of poverty [9]. Social and health systems in these countries may vary considerably from highly resources countries, such as the UK or Australia, for a wide range of reasons, including a lack of resources [8, 10]. Ideally, guidelines should be developed for care in poorly resourced contexts, and there have been developments in the global health field which have taken these inequities into account. There is an expanding literature on the use of community health workers in low-income contexts, and in what has been called task shifting or task sharing – some care tasks and interventions which would usually be undertaken by relatively highly paid specialist personnel have been shown to be effectively delivered by less qualified personnel and family members [11, 12]. These interventions may include supporting families of people with ID and linking these families to available community supports outside the formal health system. Countries which cannot afford high-cost care are generally not in a position to fund the research from which to develop optimal local practice guidelines.

The scarcity of well-designed studies which measure the efficacy and contextual-embeddedness of evidence-based treatments in low- and middle-income countries is well-documented [13]. A recent scoping review by the authors likewise pointed to a paucity of rigorous South African research to measure the effectiveness of mental health interventions for children and adults who have ID [14]. In the context of ID research, recent literature reviews have likewise found a paucity of strong evidence in family research that has focused on the delivery of interventions for children and adolescents in low- and middle-income countries [9, 11, 15].

The dearth of strong empirical evidence that attests to the efficacy of family interventions for people with ID creates a dilemma for clinicians in low- and middle-income countries. On the one hand, well-crafted guidelines have much to offer, but on the other, these guidelines may often be contextually inappropriate or not amenable to extrapolation due to other factors. Very little is known about how clinicians deal with this difficulty under real-world conditions, and after researching the ID literature we have not found any articles reporting on how ID mental health family intervention guidelines are adapted in local contexts in low- and middle-income countries. We, as clinicians and researchers, are serious about the wellbeing of persons with ID and their families globally, and it is important to explore and debate the adaptations made to knowledge and guidelines which come from wealthier countries, in lower income contexts. Given these considerations, the aim of this article is to explore the contextual application of an established set of guidelines – in this case the NICE Guideline 11 as discussed above – in a much lower-resourced context.

## Methods

In order to provide a first step in what we believe needs to be a larger conversation about the application of research evidence to clinical practice in majority world contexts, we report here on local South African practice which we have built up in relation to the NICE Guidelines on family support in the context of persons who have ID and behavior that challenges. The practice on which we report has been built by a team of psychologists in Cape Town, the capital of the Western Cape Province of South Africa, which is a relatively well-resourced context for low- and middle-income countries. The Western Cape Province has a population of approximately 6,510,300 [16, 17]. There are no good estimates of rates of ID in this province based in robust epidemiological data [10]. Assuming a population rate of ID at approximately 3%, a figure cited by Kleintjes et al. [18], we might expect the population of people in the province with ID to be approximately 195,000. There are six psychologists employed in state ID mental health services in the province, as we discuss below. This yields a ratio of psychologist to ID population of approximately 1:33,000.

However, we believe that the principles we use are transferrable to even less resourced contexts (including contexts in which there is no dedicated psychological service). Two of the authors (OC and CC) are members of that team, and another of the authors (CA) was, until recently, tasked with providing specialist oversight of the services. Both OC and CA have been employed in the service for 10 years, and CC for 3 years. OC serves as head of state clinical psychology services in ID in the Western Metropole of the Cape Town region; in terms of administrative hierarchies, there is another ID service in Cape Town. OC meets regularly with the psychologist in charge of that service, during which meetings clinical issues are discussed in detail, and LS is the research supervisor of that same psychologist. The authors of this article, therefore, are familiar with the practice trends in our context.

### The NICE guideline 11 of 2015 and behavior that challenges

Behavior that challenges has been cited as one of the most common reasons why people with ID are referred to mental health professionals at dedicated ID services [19]. Behavior that challenges refers to a set of discrete and observable behaviors that include verbal and physical aggression, self-injurious behavior, destructive behavior, overactivity, sexualized behavior, and night-time disturbance [20]. Behavior that challenges therefore encompasses descriptions of different behaviors with varying etiological bases and maintaining conditions. The following definition of behavior that challenges emphasizes the need to assess the impact of such behaviors on the person and their environment:

Behaviour of such an intensity, frequency or duration as to threaten the quality of life and/or the physical safety of the individual or others and is likely to lead to responses that are restrictive, aversive or result in exclusion. [21]

The NICE Guideline 11 of 2015 cites research, mostly controlled and uncontrolled comparative intervention studies, to provide low to medium quality evidence that cognitive behavior therapy (CBT), parental skills training and mindfulness therapy have led to significant reductions in parental stress and depressive symptoms among families of persons with ID [3]. The document recommends that caregivers should receive the following forms of intervention from services for persons with ID:

- Assistance and information that would allow caregivers access to relevant health services to obtain support for their own physical and psychological needs.
- The provision of respite services.
- Interventions aimed at providing families with easy-to-understand information about family advocacy; group interventions for families; formal evaluation of the perceived impact on families of the family member’s behavior that challenges; parental skills training to manage such behaviors; and an inclusive approach to ensure that family members are active role players in the decision-making process and implementation of strategies to manage behavior that challenges.
- If the caregiver presents with a mental health problem, services for persons with ID should either refer the carer for psychological and/or psychiatric intervention or render such services through their own organization. Psychotherapeutic family support should be evidence-based in accordance with existing NICE Guidelines [3].

## Results

### Applying the NICE guideline 11 of 2015 to the work of psychologists in a South African clinic for adults with ID and behavior that challenges: family interventions

The Western Cape Government Department of Health is tasked with the provision of health care services to more than 6 million persons who live in the Western Cape Province. Mental health services for community-based adults and children with ID are provided in two Out-Patient Clinics within two Psychiatric Hospitals situated in the Western and Eastern Metropole districts of the City of Cape Town. Both health facilities are affiliated to local universities for teaching and training purposes and together provide the only dedicated ID tertiary level mental health services in South Africa. The clinical capacity of the two clinics for children and adults with ID is limited by inadequate skilled resources and services for families of persons with ID in the primary health sector. Ideally, families should have primary access to mental health service provision for the person with ID, via the community clinics and district and other regional level health facilities. The under-resourcing of mental health services in general in South Africa results in many of those who could be served at a district and regional level, attending services at centrally based tertiary facilities. In recognition of the aforementioned service-delivery challenges, the Western Cape Department of Health is currently in the process of reconfiguring mental health services by re-allocating resources to community and district health services [22]. In addition, due to inequities in the South African health care system, those from rural areas with ID and mental health problems are under-represented in the specialized services here [10]. Referrals to the two clinics are therefore not representative of the entire group of persons with ID and behavior that challenges in the Western Cape Province.

Table 1 describes the implementation of the NICE Guideline 11 of 2015 by three psychologists within the context of local considerations in one of the two Out-Patient Clinics. The overall multidisciplinary mental health service consults with approximately 150–200 adult attendees per month.

**Table 1:** NICE Guideline 11 – Recommendations when working with carers

NICE Guideline 11: Recommendations when Working with Carers	The possible use of the NICE Guidelines in the context of our local clinic
*Work in partnership with children, young people and adults who have a learning disability and behavior that challenges, and their family members or carers, and:*	Collaborative working relationships are established with families. We are grounded in the basic principles of person-centered planning, social valorization and self-determinism.
Involve them in decisions about care.	We assess the amenability to psychological intervention of the person whose behavior challenges. Assessment is based on accepted psychological principles, e.g., the recommendations of Wright et al. [30] are followed to assess the individual’s amenability to CBT. The psychologist to whom the case is allocated will then formulate a provisional treatment plan that will be discussed in a joint meeting between the person with behavior that challenges and the primary caregiver that accompanied them to the session.
Support self-management and encourage the person to be independent.	While psychotherapy and person-centered initiatives are oriented towards the optimization of the service user’s self-direction and autonomy, family members frequently disclose a range of social and economic realities that prevent persons with mild intellectual disability in particular from achieving the levels of independence that could be reasonably expected based on individual analyses of information-processing capabilities and comorbidity (or the lack thereof). As part of a non-exhaustive list, family members have described the following challenges: Personal health and safety concerns: Even if a person with behavior that challenges has the potential to use public transport and community-resources independently, family carers seldom allow persons with ID to attend community events on their own because of Cape Town’s high levels of violent crime and the unreliability of local trains and buses. As far as we know, in the last five years only four service-users have independently made use of public transport to attend psychological services at the clinic. Poor transport infrastructure: Consistent with the findings of at least one study, parents often report negative, stigmatizing attitudes from taxi drivers when using public transport with their children [31]. Unemployment: With an unemployment rate of approximately 28%, work in the open-labor market is seldom attempted [32]. In the last five years, we have seen only two people with ID and behavior that challenges in our service who is employed in the open-labor market. This may be considered against the background of generalized neglect of vocation and occupational opportunities for adults with ID in the Western Cape Province [33]. Lack of vocational training and opportunities: With the exception of the clinic’s Occupational Therapy Day Programme, the limited number of protected employment services as well as skills and development programs do not consider applications for placement if the person presents with significant levels of aggression or self-injurious behavior. The majority of persons who access the clinic have no recourse to attend vocational services; families of persons with intellectual disability and behavior that challenges are therefore left with limited pathways to pursue increased self-direction and autonomy in the working environment.
Build and maintain a continuing, trusting and non-judgemental relationship.	All the psychologists are grounded in Rogerian principles that were attained in postgraduate training and refined in formal supervision as trainee psychologists and independent practitioners.
Provide information: About the nature of the person’s needs, and the range of interventions (for example, environmental, psychological and pharmacological interventions) and services available to them. In a format and language appropriate to the person’s cognitive and developmental level (including spoken and picture formats, and written versions in Easy Read style and different colors and fonts).	A comprehensive, detailed account of available health, educational and social services is not offered to families, in part because the geographical inequities in such services within Cape Town make it difficult to develop a uniform information package that could be shared with families, that is tailored to their own social and economic context and circumstance. Government has attempted to address this, but access to South African government-funded services continues to show geo-political imbalances long after the demise of Apartheid: as a general pattern, the areas formerly reserved for Whites have proportionally more resources than the townships that developed on the outskirts of cities, such as Cape Town and rural areas [10]. Access to private health care and education is dependent on the family’s available finances. The psychologists at the clinic therefore provide information about available services only after they have acquired information about the family’s proximity to available resources, their ability to make use of public transport to access available services and whether there are adequate finances to ensure the sustainability of the intervention. The psychologists adapt interviews according to the person’s intellectual and communication ability, e.g., language is simplified and recognized procedures are followed to assess the capacity of persons with mild and moderate intellectual disability to understand questions: - First, questions are asked without adaptation and the person’s responses are gauged. - Last, an observed failure to follow questions will result in the following adaptations that are introduced in graded fashion: Language and grammar are simplified, usually by avoiding complex sentences whilst attempting to deliberately simplify vocabulary. Because it is easier to comprehend the active form of language, questions are often signified by deliberate alterations in tone and voice inflection without reverting to the passive form of language, e.g., *You were happy (ˈ◌) to go home (↗◌)?*, as opposed to: *Were you happy to go home?* (minimal change in voice inflection). If unsuccessful, questions are presented in multiple choice format. As a final step, the person will be asked to demonstrate, either verbally or non-verbally, their preference when presented with two options. The duration of interviews or psychotherapy sessions is adjusted when persons present with prominent attention-deficits and concentration difficulties. None of the psychologists are formally trained in the use of augmentative visual communication material such as the Picture Exchange Communication System, in part because of limited resources and local training opportunities. Pictures are used informally to augment verbal instructions, and recognized psychometric tests with pictures such as the Glasgow Depression and Anxiety Scales are employed. We also make use of a website that contains hundreds of pictures that could be downloaded without incurring costs [34].
Develop a shared understanding about the function of the behavior.	As part of the collaborative therapist-family relationship, the functional intent behind behavior that challenges is explicitly acknowledged, alongside the presence of possible antecedents and maintaining conditions that are in turn associated with internal behaviors or environmental and ecological variables.
Help family members and carers to provide the level of support they feel able to.	This recommendation raises important questions when considering its application in the local context: in as much as most parents of adults with ID and severe behavior that challenges have no recourse other than the possible institutionalization of their child through certification under the Mental Health Care Act, in a poorly-fit-for-purpose ID service facility in a Psychiatric hospital, by default most of these families are required to meet their children’s high behavioral support needs at home, often with little or no formal support. In our experience, family members regularly describe perceptions of inadequacy, helplessness, and hopelessness in meeting the behavioral support needs that their family members require. Families may not have any choice other than trying to meet high behavioral support demands, regardless of the extent of their caregiving capacity and coping resources.
*When providing support and interventions for people with a learning disability and behavior that challenges, and their family members or carers:*	
Take into account the severity of the person’s learning disability, their developmental stage, and any communication difficulties or physical or mental health problems.	The Diagnostic Criteria for Learning Disabilities are used to systematically assess behavior that challenges [20]. We do not have the capacity to individually assess each referral in terms of the person’s level of intellectual disability, and some of the persons referred to us have never been psychometrically assessed. At times, the severity of intellectual disability is therefore based on clinical observation in the absence of rigorous psychometric assessment and a more comprehensive and formalized analysis of adaptive abilities and support needs. This approach is in line with the definition of ID in the Diagnostic and Statistical Manual of Mental Disorders (5th ed.) [35], in which greater diagnostic value is placed on adaptive function than previously.
Aim to provide support and interventions: In the least restrictive setting, such as the person’s home, or as close to their home as possible, and In other places where the person regularly spends time (for example, school or residential care). Aim to prevent, reduce or stop the development of future episodes of behavior that challenges. Aim to improve quality of life. Offer support and interventions respectfully. Ensure that the focus is on improving the person’s support and increasing their skills rather than changing the person. Ensure that they know who to contact if they are concerned about care or interventions, including the right to a second opinion.	The small number of psychologists has a limiting effect on their capacity to render services in the communities of service-users and their families. But as an alternative to home visits, for example, psychologists would arrange and attend person-centered meetings at vocational and residential services across the city on a regular basis. We also complete naturalistic observation and other forms of recording in the person’s vocational environment and conduct assessments such as the Functional Assessment Screening Test (FAST) with supervisors in the working environment. The FAST is relatively easy to administer and available on the internet without charge, which could potentially be used in scarce-resourced settings. Depending on the individual clinical needs of families and the person whose behavior is challenging, observations and the implementation of behavioral programs also frequently require home or workplace visits. The labor-intensive and time-consuming nature of such visits cast doubt whether such clinical practices will be sustainable in future because of an increased number of referrals. We are striving to engage in clinical processes that aim at achieving the following: The deceleration of unwanted behaviors and acceleration of target behaviors to avert, decrease or discontinue distressing forms of behavior that challenges. Such behaviors are conceptualized as being caused by the interaction between a person and other people in a dynamic environment; and helper attributions of internality are actively interrogated and discouraged in supervision. Tangible gains in the person’s quality of life. The focus is not on a skills deficit but rather on a behavioral support-needs base. Observations of inadequate medical or care support to the recipients of care often form the basis of referrals to medical professionals and social workers. Because of a shortage of appropriately qualified health care professionals, it is most often not possible to refer families for a second opinion in the governmental health services. Referrals to private health care professionals for second opinions have been made in the past, provided that the family has the financial resources to access these services.
*Advise family members or carers about their right to, and explain how to get:*	
A formal carer’s assessment of their own needs (including their physical and mental health).	A comprehensive battery of tests is available to assess aspects of parenting that include caregiving stress, depressive symptoms, coping, relationships within family context, and quality of life. While our clinic has adequate funding to purchase tests and top-up answer sheets, some of the instruments are available in the public domain and could be downloaded from the internet without additional cost implications. We believe that it is possible to conduct a formal assessment, even in settings with poorer resources, provided that there is access to the internet to download recognized assessments scales. The available assessment instruments include the following: The Beck Depression Inventory, Second Edition. Family Community Participation Survey. Family Quality of Life Survey. The OK Health Check. The Parental Locus of Control Test. The Parenting Stress Index, Short Form. The Ways of Coping Questionnaire. The Parenting Sense of Competence Test. Patient Health Questionnaire. The Self-Reporting Questionnaire. The Bene Anthony Family Relations Test. The small clinical component of psychologists implies that assessments can only be considered when parents present with severe levels of distress, helplessness and depressive symptoms. In most instances, a degree of task-shifting is manageable: parents are referred to community clinics if there are reports of physical or mental health problems; a significant minority of parents are also able to afford private health care. In both instances, we will attempt to either contact the other health care professional or compile a short written report when referring family members for assessment in the community.
Short breaks and other respite care.	Until recently the Western Cape Government Department of Health allocated 20 placements for community-based adults with ID, often with behavior that challenges, in ID Hospitals. Members of the multidisciplinary team referred to these brief admissions into hospital as “respite beds”, and the rationale of the service was to offer family brief respite from caregiving. The service in one of the two settings has since been discontinued and less than 10 placements are now available. Some of the psychologists facilitate family meetings that discuss ways of providing elderly parents with regular respite breaks, in this instance by older siblings or relatives who function as substitute caregivers in the home environment of their brother or sister.
*When providing support to family members or carers (including siblings):*	
Recognize the impact of living with or caring for a person with a learning disability and behavior that challenges.	In clinical supervision, we are encouraged to consider the role of stressful parental events juxtaposed with positive parental experiences. Case conceptualizations incorporate parental attributions, attachment theory, parental coping models and accumulative stress proliferation to unpack aspects of the child’s behavior that challenges. Such considerations are further contextualized by exploring parental roles within dynamic family systems and cultural embeddedness.
Explain how to access family advocacy.	We do not formally incorporate information-sharing initiatives aimed at fostering family advocacy or access to formal support structures to facilitate family advocacy. Substantial infringements on the rights of family members with ID and behavior that challenges require tangible interventions which may include recommendations that families should access appropriate advocacy and/or service resources that include the following local organizations. Except for the statutory Mental Health Review Board, all are non-profit organizations. The Down Syndrome Association of the Western Cape. Autism Western Cape. Cape Mental Health Society. The Down Syndrome Association of the Western Cape. The Mental Health Review Board of the Western Cape. The Western Cape Cerebral Palsy Association. The Western Cape Forum for Persons with Intellectual Disability. It is at times difficult to implement sustainable family advocacy interventions, in particular when the person with ID is denied access to educational, vocational and residential services because of their behavior that challenges. Most of these services are scarcely resourced and demand tends to far outweigh supply. Waiting lists, most often extending over one to three years, are the norm, and the majority of families that access our service have limited agency to mobilize and play an advocacy role on behalf of their family members with ID and behavior that challenges.
Consider family support and information groups if there is a risk of behavior that challenges, or it is emerging.	A recent initiative to start family support groups in the clinic yielded disappointing results. Only a small number of mothers participated, and attendance was inconsistent. Parents cited the unreliability of public transport with its relative cost implications as reasons why it was difficult to attend groups on a regular basis.
Consider formal support through disability-specific support groups for family members or carers and regular assessment of the extent and severity of the behavior that challenges. Provide skills training and emotional support, or information about these, to help them take part in and support interventions for the person with a learning disability and behavior that challenges.	Please refer to previous two paragraphs. Parental skills training is often employed after an individualized functional assessment found links between aspects of parenting and the child’s behavior that challenges. It employs the basic principles of positive behavior support and considers individual parental coping strategies and resources. We are also trying to obtain funding for parents with children with severe behavior that challenges to attend an accredited three-day course in the use of low arousal approaches in the management of behavioral difficulties.
*If a family member or carer has an identified mental health problem, consider:*	
Interventions in line with existing NICE Guidelines, or Referral to a mental health professional who can provide interventions in line with existing NICE Guidelines.	Psychotherapy is seldom offered to parents of children with ID and behavior that challenges. A parent with mental health problems is referred to public community clinics in their neighborhood. Referrals to private psychiatrists, psychologists and social workers will be considered only if the cost implications are affordable to the family. Cognitive-behavior therapy or supportive psychotherapy will be offered to parents with mental health concerns if referrals to community clinics or private health care professionals cannot be completed – in our experience community clinics differ significantly because the burden of care is not evenly distributed amongst community clinics; local organizational factors such as low staff morale could also protract the referral process. The extent of the shortage of psychologists and psychiatrists in community health services implies that mental health interventions will mostly be conducted by nurses and medical general practitioners.

## Discussion

It is important to stress that the approach discussed in this article is based largely on the authors’ own extensive clinical experience and is not based on, for example, a research study where participants are asked to respond to specific questions. This is a limitation of our work, but it is also important to point out that in terms of dedicated ID mental health services in the province in which we work, there are only six psychologists, and we work closely with all of these practitioners and act as clinical supervisors to all but one of these six psychologists. This last psychologist is supervised by a practitioner who in turn is supervised by OC.

The emerging field of global mental health makes it clear that though there are many features of evidence-based treatments which are transferable in a range of contexts, careful attention to local norms, contexts and resources are important [23–28].

The implementation of many of the NICE Guideline 11’s recommendations for carer support and family interventions is feasible in a South African context, particularly those recommendations which deal with an explicit *conceptual* recognition of collaboration, social valorization, individualization, social inclusivity and a clinical understanding of behavior that challenges as involving interactive processes between the person and his environment. We believe that the ethos of the NICE Guidelines could be applied in all settings: a collaborative working relationship, respect and treating all persons with dignity enables a level of therapist and psychologist responsivity that is independent of fiscal and human resources.

Economically South Africa is positioned between low-income and high-income countries and has been designated by the World Bank as a “higher middle-income country” [29]. The psychologists in the clinic described having access to contemporary electronic journals and research findings; we have a well-stocked psychometric test library; and it is possible to obtain funding to attend international scientific conferences. There are limited training opportunities, but internationally-accredited courses are available in some of the interventions that are recommended in the NICE Guideline 11, for example, cognitive-behavior therapy, low-arousal approaches in the management of behavior that challenges, and recognized assessment instruments such as the Autism Diagnostic Observation Schedules (ADOS). Our local clinic context is in stark contrast to services in most rural areas in low-income countries, particularly those that lack coordinated mental health care services. While we are able to successfully implement many of the NICE Guideline recommendations with regard to family interventions, a major problem is that we can only offer these services to a select few families – the majority of families in need of similar services do not have access to them because of a lack of primary health care capacity and geographical inequities in accessing health care, especially in rural areas. The effectiveness of behavioral interventions is also hampered by environmental and social factors such as poverty/low socio-economic status and unemployment. South Africa’s prevailing social inequalities and other societal issues, such as high levels of aggression and crime in communities, continue to cause family distress and, inasmuch, expressions of behavior that challenges need to be firmly contextualized within the broader socio-political context.

## Conclusions

We have argued that where guidelines such as the NICE Guidelines do not apply throughout contextually, it remains important to retain the principles behind these guidelines in local contexts. For example, in the South African context it will be salient to identify and use different pathways to facilitate respite for parents and families. The focus would not be on a respite service such as recommended by the NICE Guidelines, but rather the involvement of older siblings and other relatives as temporary caregivers in the home environment of the person with ID and behavior that challenges. However, this does not exclude recruitment of wider community resources and development of focused non-profit organizations to meet the need for respite care. It would also be important to identify how the principle of respite care is, or could be, applied in more poorly resourced contexts and countries, although this is an under-researched area.

In order to address the transferability of the NICE 11 Guidelines into different contextual situations, and taking the considerations discussed into account, we suggest that smaller descriptive, qualitative studies are necessary to explore the contextual limitations and resource strengths that exist in low- and middle-income settings. It will also be helpful to study these issues more systematically than to draw only on the clinical experience of authors, as we have done. In the absence of evidence-based research, smaller research initiatives could set the agenda by plotting ways to strengthen existing clinical, family, and community interventions whilst simultaneously building an informed foundation for future research. Of course, the need for rigorous evidence-based treatments and interventions in low-income and middle-income countries remains a priority.

## Abbreviation

ID: Intellectual disability
